# Temporins: Multifunctional Peptides from Frog Skin

**DOI:** 10.3390/ijms24065426

**Published:** 2023-03-12

**Authors:** Luca Domenico D’Andrea, Alessandra Romanelli

**Affiliations:** 1 Istituto di Scienze e Tecnologie Chimiche “G. Natta”, CNR, Via M. Bianco 9, 20131 Milano, Italy; 2 Dipartimento di Scienze Farmaceutiche, Università degli Studi di Milano, Via Venezian 21, 20133 Milano, Italy

**Keywords:** peptide, temporin, antimicrobial, mechanism, structure

## Abstract

Temporins are short peptides secreted by frogs from all over the world. They exert antimicrobial activity, mainly against Gram-positive bacteria, including resistant pathogens; recent studies highlight other possible applications of these peptides as anticancer or antiviral agents. This review is meant to describe the main features of temporins produced by different ranid genera. Due to the abundance of published papers, we focus on the most widely investigated peptides. We report studies on their mechanism of action and three-dimensional structure in model systems mimicking bacterial membranes or in the presence of cells. The design and the antimicrobial activity of peptide analogues is also described, with the aim of highlighting elements that are crucial to improve the bioactivity of peptides while reducing their toxicity. Finally, a short section is dedicated to the studies aimed at applying these peptides as drugs, to produce new antimicrobial materials or in other technological uses.

## 1. Introduction

Frogs’ skin is a rich source of bioactive compounds, such as alkaloids, biogenic amines and peptides such as neuropeptides and antimicrobial peptides (AMPs) secreted by the cutaneous exocrine apparatus [1,2]. The production of AMPs is not common to all frog families; among the *Neobatrachia*, AMPs have been identified in the *Cicroglossidae*, *Hylidae*, *Hyperoliidae*, *Leptodactylidae*, *Myobatrachidae* and *Ranidae* families, while they have not been found in *Bufonidae*, *Ceratophyridae*, *Eleutherodactylidae*, *Microhylidae*, *Pyxicephalidae* or *Rhacophoridae* [3,4]. Each frog produces its own set of peptides that may act in synergy [5]. Typically, 10 to 20 peptides belonging to different families can be found in frogs’ secretions; the composition and structure of the peptides are different within each family, including linear and cyclic peptides, disulphide bridged peptides or sequences without cysteines [6]. The diversity of secreted peptides was hypothesized to be a strategy to gain maximum protection from different microorganisms and reduce the chance of inducing resistance to individual peptides [7]. The pattern of secreted peptides can be employed as a biomarker of a particular frog population [8]. In some species, AMP production is regulated by the thyroid hormones, and may be seasonal and affected by environmental factors [9,10]. The synthesis of peptides occurs in the multinucleated cells of the dermatous skin glands. Peptides are stored in the secretory granules and released by a holocrine mechanism [7]. The physiological role of secreted AMPs is still debated, although the majority of published studies are focused on their protective function against pathogens. Studies on the activity of these peptides against pathogens present in the animal environment are scarce compared to those on pathogens involved in human diseases [4,5]. The activity of frogs’ secreted peptides was demonstrated against *A. hydrophilia*, a bacterial strain present in frog skin, for combinations of temporin A and temporin L, temporin B and temporin L, bombinin-like peptide and brevinin-2-like peptide. In addition, temporin A, esculentin-2P, dermaseptin-B1 and ranauterin-2P were found to be active against frog viruses.

The observation that AMPs are co-secreted with neuropeptides and exert a cytolytic action suggests that the so-called AMPs support the neuropeptides’ action against predators by enhancing their delivery to the endocrine and nervous systems of the predators [1].

The sequences of these peptides are different in different amphibians, and the abundance of amino acids seems to be related to the geographical location of the amphibians. While alanine is abundant in peptides from South American frogs, leucine is more abundant in peptides secreted from European frogs [11].

A phylogenetic analysis of genes encoding skin AMP peptides from *Hylidae* and *Ranidae* shows that the genes evolved from a common locus. Peptides such as caerins from Australian hylids, brevinins, ranalexin, temporins from ranin and dermaseptins from South American hylids are derived from a common precursor sequence belonging to the preprodermaseptins family. Molecular cloning from cDNA libraries obtained from skin secretions reveals that AMPs are derived from tripartite precursors containing a signal peptide, a spacer, also named the acidic propiece, and the active peptide. The signal sequence is highly conserved between related species, whereas the spacer bioactive peptide differs between anaurans species. The diversity of active peptide sequences, likely being the result of adaptation to challenges by pathogens, is such that even phylogenetically related species produce non-identical peptide sequences [12,13].

Based on the sequence, peptides are divided into 14 families: Brevinin-1, Brevinin-2, Esculentin-1, Esculentin-2, Japonicin-1, Japonicin-2, Nigrocin-2, Palustrin-1, Palustrin-2, Ranacyclin, Ranalexin, Ranateurin-1, Ranateurin-2 and Temporin.

In this review, we will focus on the temporin family. Temporins are short peptides (8–17 amino acids long), showing broad spectrum activity against several microorganisms harmful for human health. The ease with which they are obtained and modified by chemical synthesis renders these peptides ideal starting materials to produce new antimicrobial agents.

Temporins were isolated from skin secretions of frogs such as *Rana erythraea* and *Rana esculenta* for the first time [14]. In 1996, after screening a cDNA library of the skin of *Rana temporaria*, precursors of three temporin peptides (temporin B, G and H) were identified. The precursors start with a signal peptide, and the mature peptides are preceded by the Lys-Arg sequence, a processing site for convertase, and terminate with the Gly-Lys sequence, which is processed by carboxypeptidases to produce amidated C-terminal peptides. The predicted mature sequences corresponded to two 13-amino-acid peptides and one decapeptide. The three peptides, together with other similar peptides (temporin A, temporin C, temporin D, temporin E, temporin F, L and K) were isolated from skin secretions of stimulated *R. temporaria* frogs. Isolated peptides were found to be all amidated at the C-terminal end; biological assays performed on synthetic peptides revealed that all peptides, apart from temporin D and H, show antibacterial activity. Since then, more than 150 peptides belonging to the temporin family have been isolated from ranid frogs of different genera such as *Amolops*, *Hylarana*, *Lithobathes*, *Odorrana*, *Pelophylax*, *Rana* and *Hylarana* [15]. The names of peptides follow the frog species names, as reported in the literature [16]. The sampling of peptides typically occurred upon mild transdermal electrical stimulation of live frogs, which causes contraction of the adrenergic myocytes with consequent release of their content. Samples were fractionated through HPLC, tested and identified through mass spectrometry (MS) techniques. Table 1 reports temporin sequences grouped by frog genera. Temporins share the following common features: the length, most of them are 13 amino acids long; the presence of at least one basic residue (lysine or arginine); the lack of cysteine. Some peptides contain non-proteinogenic amino acids, for example, temporin M from the Russian brown frog *Rana temporaria* has an oxyproline residue in the place of the proline found in Temporin M isolated from European *Rana temporaria*. The length of peptides is variable, ranging from 8 amino acids in temporin SHf from *Pelophylax saharicus* to 17 amino acids in temporin LTc from *Hylarana latouchi* [13].

Due to the large amount of data present in the literature on temporins, we cite examples of temporins produced from the different frog genera, and we discuss in more detail peptides for which more data have been reported. Particular attention was paid to the most recent studies describing structural details along with mechanism of action studies.

## 2. Temporins from *Rana*

Peptides isolated from frogs belonging to the *Rana* genus are the most widely investigated. Here, we will discuss peptides isolated from the Chinese brown frog *Rana chensinensis* and from the European frog *Rana temporaria*. We will describe the main features of temporins 1CE, A, B, G and L and their synthetic analogues. These peptides are all active against Gram-positive bacteria, do not have a defined secondary or 3D structure in buffer and fold in the presence of bacterial membranes or bacterial membrane mimics.

### 2.1. Temporin 1CE Family

Eight temporins were isolated from a skin cDNA library of the Chinese brown frog *Rana chensinensis* [18,19] (see Table 1). Studies on the bioactivity are reported on only five peptides (Table 2).

#### 2.1.1. Temporin 1 CEa

Temporin 1CEa is a 15-amino-acid peptide, the most active among the temporin 1CEs, showing higher antimicrobial activity against Gram-positive than Gram-negative bacteria (Table 2) and significant haemolytic activity (lethal dose 50 (LD_50_) = 99 μM) [18,20,21]. Temporin 1CEa also shows anti-inflammatory activity; it binds to lipopolysaccharide (LPS), as revealed through isothermal titration calorimetry (ITC) experiments, mainly through electrostatic attraction inhibiting the inflammatory effects of LPS [20]. The dissociation constant value of temporin 1CEa for LPS is around 0.1 µM. Temporin 1CEa downregulates the MyD88-dependent signalling pathway, reducing the release of proinflammatory molecules as tumour necrosis factor-α (TNFα) and interleukin-6. The peptide has an interesting anti-cancer (antiproliferative) activity, with IC_50_ ranging from 30 to 60 μM against several cell lines [21]. It is particularly active against human breast cancer cells. Transmission electron microscopy (TEM) analysis revealed that in one hour, the peptide causes membrane breakage with leakage of intracellular components. In contrast, no damage to erythrocytes was observed at 50 μM and no cytotoxicity was observed against normal human endothelial cells. Studies in human A375 melanoma cells showed that Temporin 1CEa targets phosphatidylserine (PtdSer) overexpressed on the cancer cell surface. Temporin 1CEa binds PtdSer, as assessed through ITC, and this interaction elicits peptide helical structure favouring the membrane binding and inhibitory activity [22].

The molecular mechanism at the base of the anticancer activity of temporin 1CEa was analysed in different human breast cancer cell lines [23]. The peptide binds to cell membranes, disrupting membrane integrity, inducing permeability and causing the release of intracellular Ca^2+^. Scanning electron microscopy (SEM) and TEM studies showed that peptide at high concentrations disrupts cell membranes, inducing depolarization. In this condition, the peptide is internalized and targets the mitochondrial membrane (which is negatively charged), triggering mitochondrial membrane depolarization and reactive oxygen species (ROS) overproduction. Temporin 1CEa is unordered in water and assumes a helix structure in 50% trifluoroethanol (TFE)/water mixture [21].

#### 2.1.2. Temporins 1-CEb, 1-CEc, 1-CEh

Temporin 1CEb is a 12-mer peptide with an overall 1+ charge (also known as amurin-3 from *Rana amurensis*). This peptide is active against Gram-positive bacteria and less active against Gram-negative bacteria, and it presents significant haemolytic activity (LD_50_: 112 μM) [18]. It is unordered in water, but it assumes a helical structure in the presence of 50% TFE or 30 mM sodium dodecyl sulphate (SDS), suggesting that this conformation could be assumed when the peptide interacts with bacterial membranes. Temporin 1CEb was the starting point to develop more potent analogues, as described below.

Temporin 1CEc has low antibacterial activity and also low haemolytic activity (HC_50_ ≥ 500 μM). For this reason, it inspired the design of new analogues (see infra).

Very recently, a new Temporin 1CE family member was identified and characterized: Temporin 1CEh [24]. The peptide shows inhibitory activity, mainly against Gram-positive bacteria, but it is endowed with an elevated haemolytic activity (HC_50_ = 152.6 μM).

**Table 2:** Antimicrobial activity against selected microorganisms of peptides from *Rana chensinensis*.

Peptide	Antimicrobial Activity as MIC (µM)			
	*S. aureus 22401*	Ref	*E. coli 44102*	Ref
Temporin 1CEa	14.4	[18]	>100	[19]
Temporin 1CEb	41	[18]	>100	[18]
Temporin 1CEc	>100	[18]	>100	[18]
Temporin 1CEe	6.250	[19]	50.0	[19]
Temporin 1CEh	8	[24]	128	[24]

MIC = minimum inhibitory concentration.

#### 2.1.3. Temporin 1 CEa Analogues

Temporin 1CEa analogues were designed with the aim of improving anti-cancer activity against human breast cancer cells and specificity while reducing haemolysis [25]. Hydrophobicity and cationicity were modulated by introducing Lys and Leu residues on the polar and apolar surface of the peptide, respectively. All designed peptides are unordered in water but fold in a helical conformation in the presence of 50% TFE or SDS. Structure–activity studies revealed that increasing the peptide positive charge enhances cancer cell specificity, whereas moderate hydrophobicity is required for reducing the haemolytic activity. Six analogues were tested, and the peptide LK3 (FKKLKKIANIINSIFKK-NH_2_), with lysine residues in position 2, 3 and 16 (in place of valine, aspartic acid and glycine), emerged as the most interesting, being more active than the parent peptide with an inhibitory concentration (IC)_50_ of 27.72 μM (MCF-7 cell line) and 25.04 μM (Bcap-37 cell line) and lower haemolytic activity, with an LC_50_ of 4.468 µM vs. the 95.74 µM of temporin 1CEa.

#### 2.1.4. Temporin 1CEb Analogues

Temporin 1CEb analogues were designed to obtain a novel compound with improved bioactivity against pathogens and reduced haemolytic activity [26]. The primary amino acid sequence was modified in order to increase the overall positive charge (introducing Lys) and decrease hydrophobicity to modulate haemolysis. In particular, the analogue L-K6 (KKILSKIKKLLK-NH_2_) presenting a 7+ charge was very promising. It was more active than the parent peptide against several Gram-negative and Gram-positive bacteria (minimum inhibitory concentration (MIC) = 3.25 μM) and almost not haemolytic (LD_50_ > 1000 μM). The L-K6 peptide assumes a helical conformation in membrane-like environments such as in the presence of SDS (30 mM) or TFE as a cosolvent (50%). The peptide enhanced the permeability and induced the depolarization of the bacterial membrane. This peptide showed promising application in the treatment of dental caries, being able to kill oral pathogens such as *Streptococcus mutans* (MIC: 3.13 μM) as well as *Candida albicans* (MIC: 6.25 μM) [27]. More interestingly, L-K6 was also able to inhibit biofilm formation and reduce the biomass of an existing biofilm of *S. mutans*; furthermore, it could decrease the inflammatory response mediated by LPS, modulating the MyD88-dependent pathway and inhibiting the expression of pro-inflammatory molecules [28].

The role played by single or multiple tryptophan residues introduced in place of the apolar amino acids of the L-K6 peptide was also evaluated [29]. Analogues with a single-point mutation (I1W and I4W) were endowed with improved antibacterial activity, especially against Gram-negative bacteria, such as *Klebsiella pneumoniae*, *Enterobacter cloacae* and *Proteus mirabilis*; in contrast, the peptide L-K6 is inactive. The presence of tryptophan does not affect the haemolytic activity of this class of peptides. The introduction of a tryptophan residue in the N-terminal region increases the affinity for negatively charged lipids, with the side chain of the tryptophan inserting into the bilayer as demonstrated by fluorescence and quenching studies. The penetration of the tryptophan side chain into the membrane improves the ability of the peptide analogues to break the negatively charged liposomes; this could explain the ability of these tryptophan analogues to kill bacteria. In addition, the replacement of leucine in position 5 with tryptophan (L5W) mainly improves the antibacterial activity against Gram-positive bacteria. Finally, the introduction of two tryptophan residues in the analogue L-K6 afforded peptides I1WL5W and I4WL5W, featuring strong antimicrobial activity against both Gram-positive and Gram-negative bacteria and low cytotoxicity [30]. The mechanism of action of these peptides consists of binding to the bacterial membrane, as suggested by fluorescence studies, mainly due to electrostatic interactions, followed by the insertion into the lipid phase of the aromatic side chains, which triggers membrane destruction. These results were further confirmed through SEM analysis on *Escherichia coli* and *Staphylococcus aureus* bacteria.

The peptide L-K6 exhibits anticancer activity, especially against MCF-7 cancer cells [31]. The mechanism of action consists of a selective binding to the cancer cell surface, determined by the presence of PtdSer, and internalization mainly via a clathrin-independent macropinocytosis, causing nuclear damage without affecting the cytoskeleton, as revealed using super-resolution microscopy. Inside the cancer cell, L-K6 likely targets the DNA, inducing chromatin decondensation and DNA double strand breaks. In a subsequent in vitro study, it was shown that L-K6 induces sensitivity to doxorubicin in resistant breast cancer cells MCF-7/ADR, suggesting a possible combined use of L-K6 and chemotherapeutic agents to overcome drug resistance in cancer chemotherapy [32].

Another interesting analogue of temporin 1CEb is the peptide D-K5 described by Shang and co-workers, derived from L-K6, with a D-Lys in position 7 [26]. It was selected to prepare a bifunctional molecule presenting the antibacterial peptide D-K5 linked through a PEG spacer (8-amino-3,6-dioxaoctanoic acid) to a Leu-enkephalin analogue (dalargin, DAL), a peptide with cytoprotective and pro-proliferative activity for application in infected wound healing [33]. Conjugation affects the antimicrobial properties of the original peptide to a small extent; however, the bifunctional peptide shows significant cytotoxicity towards keratinocytes and fibroblasts when used at higher concentrations. Successively, the immunomodulatory properties of the conjugate DAL-PEG-DK5 were studied [34]. In vitro analyses demonstrated that the DAL-PEG-DK5 peptide shows anti-inflammatory activity against LPS-induced inflammation. The peptide interacts with LPS, and this interaction drives peptide aggregation, which hampers LPS recognition by the host cell, thereby inhibiting intracellular signalling and decreasing the expression of pro-inflammatory cytokines. In vivo experiments showed that the DAL-PEG-DK5 peptide protects mice from septic shock mediated by LPS. Finally, it was shown that this peptide exerts antimicrobial activity against methicillin-resistant *Staphylococcus aureus* (MRSA) [35]. In particular, DAL-PEG-DK5 showed good bacteriostatic activity against several MRSA strains isolated from infected patients, with an MIC of 40 μg/mL against the *S. aureus* USA300 strain. Furthermore, the peptide was also able to disrupt the biofilm of the USA300 strain. The mechanism of action consists of bacterial membrane permeabilization and disruption. The peptide is able to accumulate in the cytoplasm of infected human keratinocytes, leading to a significant reduction (71%) in intracellular MRSA, suggesting a potential application for the treatment of *staphylococcal* skin infections.

#### 2.1.5. Temporin 1CEc Analogues

Temporin 1CEc analogues were designed to improve natural peptide antimicrobial activity, modulating the total positive charge and hydrophobicity of the peptides [36]. Structure–activity relationship studies showed that for this peptide series, a balance between hydrophobicity and cationicity is required to obtain improved biological activity. Peptide analogues 2K2L (IIPLPLKKFLKKL-NH_2_) and 2K4L (IILLLLKKFLKKL-NH_2_) have broad spectrum antibacterial activity with low or moderate haemolytic effect. Interestingly, they are also active against multi-drug resistant bacteria such as *Staphylococcus epidermidis* (MRSE1208). In this case, they inhibit biofilm formation. The peptides achieve antibacterial activity, disrupting the bacterial cell membrane as revealed through SEM and confocal laser scanning microscopy experiments on MRSE1208. The analysis of membrane permeability in the presence of the two peptides suggested the formation of pores. Finally, an analogue of temporin 1CEh named T1CEh-KKPWW (KKWVPWKKIANIL-NH_2_) was reported. It presents improved antibacterial activity against Gram-negative bacteria and low haemolysis [24].

### 2.2. Temporin A

#### 2.2.1. Temporin A Biological Activity

The temporin A peptide was isolated from *Rana temporaria* in 1996 [37]. The biological activity of this peptide is quite broad. In fact, temporin A is active against bacteria, fungi and protozoa and, in addition, it has been shown to present anticancer and insulinotropic activity (Table 3). Temporin A is mainly active against Gram-positive bacteria, such as *S. aureus* and *Bacillus megateriurn*, specific *E. coli strains* and clinically important strains such as methicillin-resistant *S. aureus* and vancomycin-resistant *Enterococcus faecium* and *Enterococcus faecalis*. The measured MIC values against these bacteria range from 2.5 to 20 µM [38]. More importantly, temporin A presents low lytic activity against human red blood cells (LC > 120 μM).

Several studies have been performed on MRSA strains, specifically to treat superficial wounds [39]. In this regard, it has been shown that temporin A, in addition to its antibacterial properties, can stimulate the healing of tissues. In fact, it reduces the number of *S. aureus* cells inside HaCaT keratinocytes, but at the same time induces the migration of HaCaT keratinocytes mediated by EGFR [40]. In vivo experiments show that temporin A is effective in the treatment of MRSA-infected surgical wounds in mice; when topically administered, it promotes healing [41]. Temporin A was investigated as a prophylactic agent in infections from *Staphylococci* [42]. Finally, temporin A inhibits biofilm formation on polystyrene surface and central venous catheters, also in synergy with a cecropin A-melittin hybrid peptide (CA(1–7)M(2–9)NH2) [43].

In addition, temporin A activity has been studied in combination with other antibiotics compounds, very often showing remarkable synergism. For example, the peptide showed synergy with imipenem (against *Pseudomonas aeruginosa*), ceftazidime (against *P. aeruginosa, S. aureus*), polymyxin E (against *P. aeruginosa*) and with linezolid against *S. epidermidis* ATCC 12228 in a subcutaneous rat pouch model [44,45]. Moreover, it is active against *E. faecalis*, also in combination with co-amoxiclav and imipenem [44]. It improves survival in a mouse model of staphylococcal sepsis when administered in combination with imipenem [46]. Temporin A enhances the antibacterial activity of gentamicin against *S. aureus* and, to a lower extent, against *P. aeruginosa* biofilm, suggesting application as adjuvant agent in the treatment of biofilm-associated infections [47]. Temporin A can also act in combination with other temporin peptides, such as a temporin B analogue or temporin L, against different Gram-positive and Gram-negative bacteria [48,49]. Notably, synergism with a modified temporin B (TB-YK) has been reported. In particular, the peptide mixture showed antimicrobial and anti-inflammatory activity in vivo against Gram-positive (*S. aureus*, *Listeria monocytogenes*) and Gram-negative (*Salmonella enterica*, *E. coli*) bacteria [49]. Temporin A is active against fungi such as *C. albicans* and channel catfish virus and the non-enveloped frog virus-3 [50]. A computational study revealed that the peptide could interact with Middle East respiratory syndrome coronavirus (MERS-CoV) protein and, hence, it could have a role in the treatment of this pathology [51]. Temporin A has strong antiparasitic action against protozoa of the *Leishmania* genus, triggering membrane destruction. For this reason, it should prevent the pathogen from developing resistance [52,53]. Furthermore, it is a chemoattractant for phagocytic leukocytes through its interaction with the Formyl Peptide Receptor-Like 1 receptor, suggesting a role in host immune response to infection [54]. Finally, temporin A showed interesting activity against human cells. It was demonstrated that it presents anticancer activity towards the non-small cancer lung cell (NSCLC) lines, and was able to induce insulin release from rat- and human-derived beta cells with potential use for type 2 diabetes treatment [55,56]. The interaction between temporin A and A549 cells, lung-derived cells, was studied through scanning ion conductive microscopy, revealing inhomogeneous binding of temporin A at the cell surface [57].

#### 2.2.2. Temporin A Structure and Mechanism of Action

The mechanism of action of temporin A is based on the molecular interaction with the bacterial cell membrane. The peptide in water is unordered, and it assumes a helical conformation when dissolved with a cosolvent (such as TFE) or a detergent (SDS), suggesting that this will be the active conformation in the presence of bacteria. The positively charged peptide is attracted by the negative charges exposed on the bacterial membrane, and the change from water to a more hydrophobic environment induces helix stabilization in the amphipathic peptide. Structural studies in SDS micelles showed that the peptide is located at the micelle–water interface; in contrast, in the presence of dodecylphosphocholine (DPC) micelles, temporin A does not fold and is not able to penetrate the hydrophobic core, suggesting a molecular explanation for its low haemolytic activity. Increasing the concentration of the peptide on the bacterial membrane, the peptide starts to assemble and destroys the membrane through a carpet-like mechanism [53,58]. In the presence of LPS micelles, temporin A assumes an oligomeric helical conformation; this could explain the limited ability of the peptide to permeate the membranes of Gram-negative bacteria, explaining its low antibacterial activity [59]. Temporin L prevents the LPS-induced self-association of temporin A; this observation suggests an explanation of the synergism and activity of the peptide mixture against Gram-negative bacteria [60].

#### 2.2.3. Temporin A Analogues

Several analogues have been designed to understand structure–function relationships and to develop a more potent antimicrobial agent. Single or double mutants, dimeric, peptides with inverted chirality (all-D) and retro-inverse analogues have been studied, but no significant advantages with respect to the natural peptide have been found [15].

### 2.3. Temporin B

#### 2.3.1. Temporin B Biological Activity

Temporin B is a 13-amino-acid peptide isolated from the skin of the European frog *Rana temporaria*. It is mainly active against pathogens such as Gram-positive bacteria, including *S. aureus*, *B. megaterium* and *Streptococcus pyogenes*, and also against fungi such as *C. albicans* (Table 4). Experiments carried out with bacteria isolated from the natural flora of the frogs reveal that temporin B is strongly active against *Acinetobacter junii* Bo-2 [61]. Temporin B effectively kills ATCC-derived and multidrug-resistant clinical isolates of *S. aureus* in HaCaT keratinocytes without injuring host cells. Interestingly, the peptide exerted a wound-healing effect when tested on keratinocyte monolayers [40]. At very high concentrations, it shows activity against *E. coli*; the activity increases when tested against *E. coli* strain mutants with defective cell walls. Antibacterial assays against an MDR clinical isolate of *P. aeruginosa* show a lack of activity in vitro, while in experiments on *Caenohabditis elegans* infected with the same bacterium, the peptide was found to be able to reduce the lethality of infected nematodes [62].

The peptide acts in synergy with temporin L against Gram-negative bacteria such as *E. coli*, *P. aeruginosa* and *Aeromonas hydrophyla*. The synergic action assumes a physiological significance and explains why the same organism produces multiple forms of AMPs.

The biological activity of temporin B was also investigated against viruses, cancer cells and parasites. The peptide displays anti-*Leishmania* activity at a micromolar concentration; it was hypothesized that it permeates the plasma membrane of the parasite [46]. Recent experiments show that temporin B inhibits herpes simplex virus 1 (HSV-1) infection in human epithelial cells. The peptide disrupts the viral envelope, as suggested in TEM experiments. In addition, it inhibits the attachment and the entry of the virus into host cells [64]. The lack of cytotoxicity and haemolytic activity render this peptide interesting for future therapeutic applications.

#### 2.3.2. Temporin B Structure and Mechanism of Action

The secondary structure of the peptide was investigated through CD spectroscopy in different conditions. In buffer at neutral pH, the peptide does not show conformational preferences; in the presence of 30% TFE or negatively charged micelles composed of SDS and 1-stearoyl-2-oleoyl-sn-glycero-3-phosphocholine (SOPC)/1-palmitoyl-2-oleoyl-sn-glycero-3-phosphoglycerol (POPG) large unilamellar vesicles (LUV), the peptide assumes a helical conformation [54,61,65]. Interestingly, when the peptide is incubated with either *E. coli* LPS or *E. coli* cells, there is no indication of a preferred conformation [60,66]. NMR studies of temporin B in LPS micelles suggest oligomerization of the peptide, involving residues at the N- and C-terminal ends [67]. When temporin L and temporin B are combined, temporin B appears as a monomer. Based on these results and on fluorescence experiments, it was hypothesized that temporin L prevents the aggregation of temporin B within LPS, therefore allowing translocation of the peptide across the outer membrane to reach the cytoplasmic membrane [60,67]. The structure of temporin B in LPS micelles was determined employing data collected on the mixture of temporin B plus temporin L. The peptide assumes a helical conformation, exhibiting a large hydrophobic face comprising residues Leu1, Ile4, Val5, Leu8 and Leu12, and on the opposite side, the two polar residues (Asn7 and Ser11), along with Lys 10. Saturation transfer difference (STD) NMR experiments indicate that only N-terminal hydrophobic residues and polar residues establish interactions with LPS, supporting the hypothesis that the self-association of the peptide by non-polar residues might be responsible for the limited interactions of the peptide with LPS micelles [67].

Initial attempts to understand the mechanism of action of temporin B were performed by studying the interactions of the peptide with lipids as model biomembranes. The peptide causes lysis of zwitterionic or acidic phospholipids, as revealed by leakage experiments with phosphatidylcholine (PtdCho), phosphatidylserine (PtdSer) and phosphatidylglycerol (PtdGro) [61]. Studies on LUV composed of SOPC and SOPC/POPG by circular dichroism reveal that the peptide folds into an α-helix upon interaction with the vesicles. Using temporin B conjugated to the fluorescent probe Texas red through an extra cysteine residue at the N-terminus, the formation of amyloid-like fibres was observed in the presence of SOPC/POPG micelles but not phosphatidylcholine (PC) [65]. The insertion of the peptide into lipid monolayers located at air/water interfaces was investigated by measuring the change in surface pressure upon injection of the peptide into SOPC/POPG micelles [68]. Temporin B penetrates monolayers, and penetration increases with increasing POPG content. The interaction of temporin B with lipids causes lipid lateral segregation and the formation of lipid-enriched microdomains. Electrostatic interactions between the positively charged peptide and the negatively charged membranes reduce repulsion between peptides, resulting in their aggregation in the membrane. Experiments with giant unilamellar vesicles (GUV) containing SOPC and POPG reveal that the peptide rapidly aggregates on the surface of the membrane and does not cause GUV dissolution [69]. This observation supports the hypothesis that temporin B destabilizes the membrane and unlike a detergent, it does not destroy it, nor forms pores. Studies on glass-supported phospholipid bilayers revealed that the interactions of temporin B with these model membranes results in the segregation of membrane-bound peptides and formation of lipid fibrillar protrusions [70], suggesting that the interaction of peptides with lipids drives membrane shape transformations. Recent studies performed using all-atom and coarse-grained molecular dynamics simulations aimed at investigating the interaction of temporin B with mixed zwitterionic and anionic phospholipids membranes support previous experimental results, confirming the ability of temporin B to produce clusters on the membrane surface and promote the extrusion of lipids [71].

The interaction of temporin B with *E. coli* LPS was investigated through ITC; in physiological conditions, i.e., at 37 °C, when the LPS is in the liquid crystalline phase, the interaction of the peptide with LPS O26:B6 is an endothermic entropically driven process, likely involving the disruption of hydrogen bonds in ordered water molecules around lipid A [72]. The titration of LPS O111:B4, which has longer carbohydrate chains as compared to O26:B6, is instead an exothermic process in the same conditions, driven by electrostatic interactions. Depending on the prevalence of hydrophobic vs. electrostatic forces, the interaction of peptides with LPS will be enthalpically or entropically driven. The ability of temporin B to interact with LPS was also proven in fluorescence experiments. Studies performed on a rhodamine-labelled and also on a dansyl-labelled temporin B in the presence of *E. coli* LPS show that the peptide self-associates in these conditions [60]. Binding studies of temporin B to LPS are reported in different papers. Binding stoichiometry studies revealed that an excess of peptide binds to LPS micelles; the dissociation constants of nitrobenzodiazole (NBD) and dansyl-labelled temporin B were comparable and found to be in the micromolar range [67,73]. Binding studies of the peptide to *E. coli* cells revealed a lack of interactions, consistent with the lack of activity against *E. coli*. TEM studies carried out on *E. coli* cells incubated with temporin B revealed no changes in the bacterial cell morphology. Overall, these data indicate that the ability of temporin B to establish interactions with *E. coli* LPS is not necessarily related to its ability to kill bacterial cells [73].

#### 2.3.3. Temporin B Analogues

Extensive work has been performed on the development of temporin B analogues. The introduction of positively charged amino acids results in a broader spectrum of action of the peptide. In 2009, Capparelli and co-workers reported an analogue modified by a tyrosine and two lysines at the N-terminus (TB-YK) [54]. This analogue was active against Gram-positive and Gram-negative bacteria. Experiments in vitro and in vivo demonstrated that this peptide acts in synergy with temporin A, in conditions that closely mimic bacterial infections in humans. Alanine scanning studies, aimed at understanding the amino acids crucial for the activity of temporin B, revealed that Lys in position 10 is fundamental for the activity of the peptide, and that substitution of the glycine residue in position 6 by an alanine resulted in a peptide with slightly improved activity against Gram-positive bacteria [63]. The introduction of two lysines at the N-terminus of the peptide in combination with the replacement of glycine by alanine resulted in a peptide (TB_KKG6A) also showing activity against the Gram-negative bacteria *E. coli* and *P. aeruginosa* [74].

**Figure 1:**
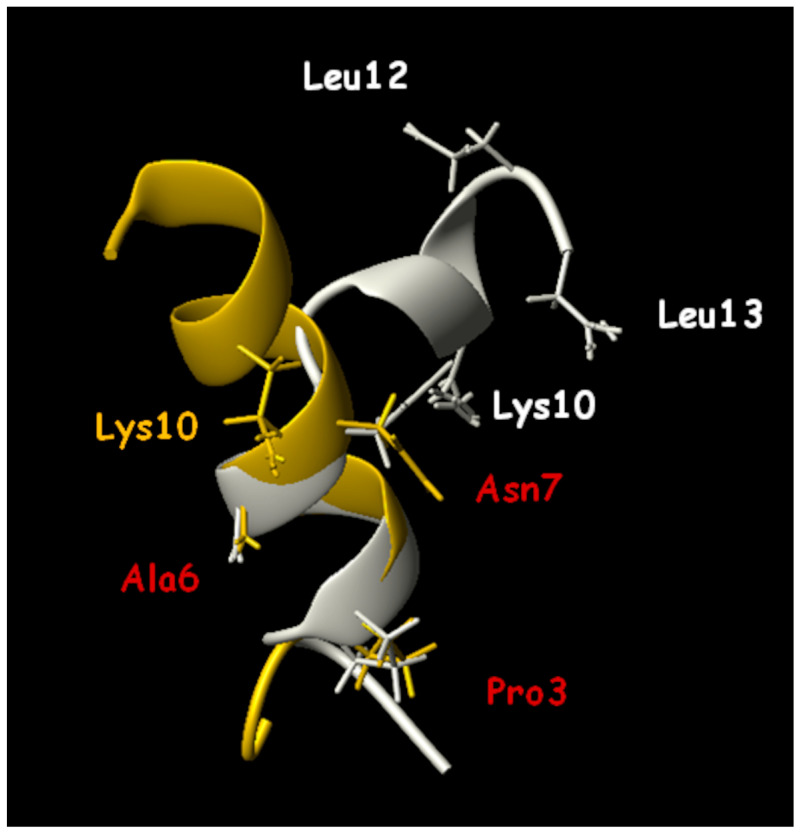
Superposition of the two structures of peptide TB_KKG6A, analogue of temporin B, obtained in the two different systems. In light grey, the structure in LPS; in gold, the TB_KKG6A structure obtained in the cellular context. The interacting residues that assume very similar orientations in the two environments are indicated in red. Reprinted with permission from Ref. [66]. Copyright 2015 American Chemical Society.

Structural studies on the peptide TB_KKG6A were carried out through NMR in the presence of *E. coli* LPS and *E. coli* cells; these studies provide high-resolution molecular details of the interaction between the peptide and bacteria. The peptide aggregates in solution and becomes monomeric when it establishes interactions with the bacterial outer membrane. A transition from a random coil to a helical structure is observed either with LPS or with cells of *E. coli*, but the three-dimensional structure in the two conditions is different: TB_KKG6A is a kinked helix when it interacts with isolated LPS, while it is an uninterrupted helix when it interacts with *E. coli* cells (Figure 1). In the latter case, the helix shows a hydrophobic face containing Val5, Leu8, Leu 9 and Leu 12, and a hydrophilic face comprising residues Asn7, Lys10 and Ser11. The N-terminal end with two lysines is mostly disordered. STD experiments revealed that amino acids on the hydrophilic face are in contact with the LPS. Altogether, structural studies suggest that positively charged residues at the N-terminus are responsible for anchoring the peptide to the negatively charged phospholipids, and Pro3, Ala6, Asn7 and Lys10 specifically bind to the membrane. Binding studies of the peptide in the presence of *E. coli* LPS and *E. coli* cells revealed that the peptide binds to LPS and to cells with a very high affinity; the stoichiometry of binding to cells is about 1 × 10^6^ peptides per bacterial cell, suggesting that peptides cover the cell membrane. The analysis of the morphology of *E. coli* cells incubated with TB_KKG6A through TEM shows the formation of cellular filaments. One hypothesis is that membrane disgregulation causes the inhibition of cell division; the process of cell division is, in fact, mediated by the formation of a “Z ring”, composed of polymerized FtsZ protein, at the site of division. If the protein FtsZ is not associated with the membrane, Z rings are not functional for division, and long filaments of cells are observed [75].

Further studies on temporin B analogues in which the amino acids in position 3, 6 and 7 were modified allowed the identification of the analogue TB_KKG6K with improved activity toward Gram-positive and Gram-negative bacteria. TB_KKG6K inhibits the growth of human pathogenic yeast and kills planktonic and sessile cells of *C. albicans* [76,77]. The introduction of D-lysines in place of L-lysine in TB_KKG6K to increase the stability to proteolytic degradation did not result in a substantial change in the peptide antimicrobial activity. Interestingly, this peptide was better tolerated than its L-lysine counterpart in vitro, in primary human keratinocytes and in 3D human epidermal models. Studies aimed at understanding the fungal cell membrane activity of TB_KKG6K were carried out using model membrane systems. The peptide preferentially interacts with anionic membranes; fluorescence microscopy experiments carried out on a fluorescein-labelled peptide and with a lipophilic dye FM4-64 suggest that the peptide compromises the integrity of the membrane and is rapidly taken up by cells through an energy-independent mechanism. The peptide does not cause cell lysis but triggers the disintegration of subcellular structures. These results suggest the existence of an intracellular target of the peptide.

A different temporin B analogue named TB_L1FK was designed through a statistics-based computational strategy, aimed at obtaining peptides showing physico-chemical features such as hydrophobicity and net charge similar to the natural peptide temporin B, and low cytotoxicity. TB_L1FK was obtained by replacing the N-terminal leucine with a phenylalanine and the C-terminal leucine with a lysine, and deleting Asn7 [78]. The antimicrobial activity of this peptide was investigated together with that of TB_KKG6A. The two peptides reduced the ability of *S. aureus* to form biofilm, while only TB_KKG6A reduced the biomass of *P. aeruginosa* biofilms. Both peptides in combination with EDTA caused a reduction in colony-forming unit (CFU) number in preformed biofilms of *S. aureus* and *P. aeruginosa*. Interestingly, no haemolytic activity was detected on human red blood cells, and TB_L1FK showed a lower cytotoxic activity against human epithelial cells.

### 2.4. Temporin G

Temporin G is a 13-amino-acid peptide isolated from the skin of *Rana Temporaria* [37]. Its antimicrobial activity was initially investigated against nosocomial multidrug-resistant strains; it exerts bactericidal activity against Gram-positive bacteria such as *E. faecium* and *S. aureus* and Gram-negative bacteria as *Stenotrophomonas maltophilia* and *Acinetobacter baumannii* at low concentrations (Table 5) [79]. The activity of the peptides is strongly reduced or lost when tested in human serum.

Temporin G can eradicate *S. aureus* biofilms; fluorescence experiments performed using the membrane-impermeable dye Sytox green revealed that the peptide induces the perturbation of *S. aureus* biofilm membranes [81]. When used in combination with tobramycin, a commercially available antibiotic, the peptide at a sub-inhibitory concentration enhances the activity of the antibiotic. Tests on human immortalized keratinocytes show that the peptide is slightly cytotoxic only at high concentrations, causing a reduction in metabolically active cells. The activity of the peptide was also tested on fungal strains: MIC50 between 4 and 16 µM were found against *C. albicans*, while a reduced activity was detected against non-*Candida* species [80]. The peptide acts by destabilizing the cell membrane structure, likely causing the formation of small local breaks. It is also effective in inhibiting the yeast-to-hyphal transition, and hampers the formation of biofilms in *Candida*. Finally, the peptide is active against filamentous pathogenic fungi such as *Microsporum gypseum* or *Trichophyton mentagrophytes*. Recently, the antiviral activity of temporin G was explored [82]. The peptide inhibits the early stages of the PR8A influenza virus replication in A459 cells; it was hypothesized that the peptide interacts either with the subunit HA1 of the protein haemagglutinin and this leads to the inhibition of the attachment of the virus to the host cell, or with the HA2 subunit and terminal regions of HA1 to prevent the conformational change of HA essential for the endocytosis of the virus into the host cells. Finally, temporin G inhibits the replication of Herpes simplex virus type 1, affecting the early stages of the virus life cycle and virion as well. The peptide triggers a reduction in infection by John Cunningham polyomavirus (JCPyV) [83]. The peptide stimulates insulin release from 1.1B4 human-derived pancreatic β cells, without cytotoxicity [56]. Experiments in mice show that temporin G injection improves glucose tolerance and causes an increase in insulin secretion, suggesting its potential application in the treatment of type 2 diabetes.

No analogue has been reported so far to the best of our knowledge.

### 2.5. Temporin L

#### 2.5.1. Temporin L Biological Activity

Temporin L is a 13-amino-acid peptide isolated from *Rana Temporaria* [37]. It is active against Gram-positive bacteria and the fungus *Candida* (Table 6). In contrast to most of the natural temporins, it also shows activity against Gram-negative bacteria. Temporin L at a subMIC concentration inhibits biofilm formation in *Pseudomonas fluorescens* in static and dynamic conditions and is able to perturb preformed biofilm architecture [84,85]. In order to investigate the antiendotoxic effect of temporin L, its interaction with LPS was investigated in vitro and in vivo. Fluorescence displacement assays revealed that the peptide binds to purified *E. coli* LPS and lipid A and penetrates into LPS monolayers. The antimicrobial activity of the peptide against *E. coli* is reduced in the presence of LPS, confirming the affinity of temporin L toward LPS. In vivo experiments on rat models indicate that treatment with a combination of temporin L and imipenem and piperacillin reduces the plasma levels of the endotoxin and TNF-alpha, resulting in high survival rates of the animals [86].

Temporin L causes lysis of human erythrocytes, with an efficiency higher than temporin A and B. It is also cytotoxic to cancer cell lines such as Hut-78, U-937 and K562 [87]. Cell sensitivity to the peptide is dependent on the cell line, but the kinetics of killing are very rapid in all cases. K-562 and U-937 cells were more resistant as compared to Hut-78.

The antiviral activity of temporin L was investigated against different viruses, including enveloped, naked, DNA and RNA viruses. The peptide is active against herpes viruses, paramyxoviruses, influenza virus and coronaviruses, including SARS-CoV-2, showing a reduced or null activity against nonenveloped viruses [85]. Based on these results, it was hypothesized that the activity of the peptide is related to its ability to interact with the viral membrane.

#### 2.5.2. Temporin L Structure and Mechanisms of Action

To understand its mechanism of action against bacteria, membrane permeabilization studies were performed by detecting the release of fluorescent dyes from lipid vesicles of different compositions, charges and sizes, such as the electrically neutral PC or PC/cholesterol and the negatively charged PC/POPG. The peptide interacts preferentially with neutral zwitterionic vesicles and perturbs membrane integrity; leakage of large dyes occurs to a lesser extent compared to that of small molecules, suggesting the formation of local pores or breaks in the membrane rather than its disruption by a detergent-like mechanism. It was hypothesized that temporin L induces a profound reorganization of the bilayer, establishing contacts with the lipid chains. In vesicles containing acidic phospholipids, the formation of clusters with lipid-bound peptide molecules is observed [68,87]. Using fluorescence spectroscopy, exploiting the tryptophan present in temporin L, Zhao and co-workers investigated the interaction of the peptide with SOPC/cholesterol, SOPC and SOPC/POPG liposomes [88]. The interaction of temporin with liposomes determines a blue shift in the emission of the tryptophan and fluorescence quenching, suggesting that the peptide is partially inserted into the liposomes. The peptide binds to SOPC liposomes and interactions strengthen in the presence of POPG. The results of these experiments support the hypothesis that the peptide interacts with the lipid bilayer in a defined fashion and does not induce significant lipid segregation, suggesting a barrel–stave mode of interaction with the bacterial membrane. Experiments carried out in parallel on temporin B and L on supported phospholipid bilayers composed of PC and PG indicate the formation of lipid tubular protrusions, suggesting that peptide–lipid interactions play a key role in membrane shape transformation [70].

**Figure 2:**
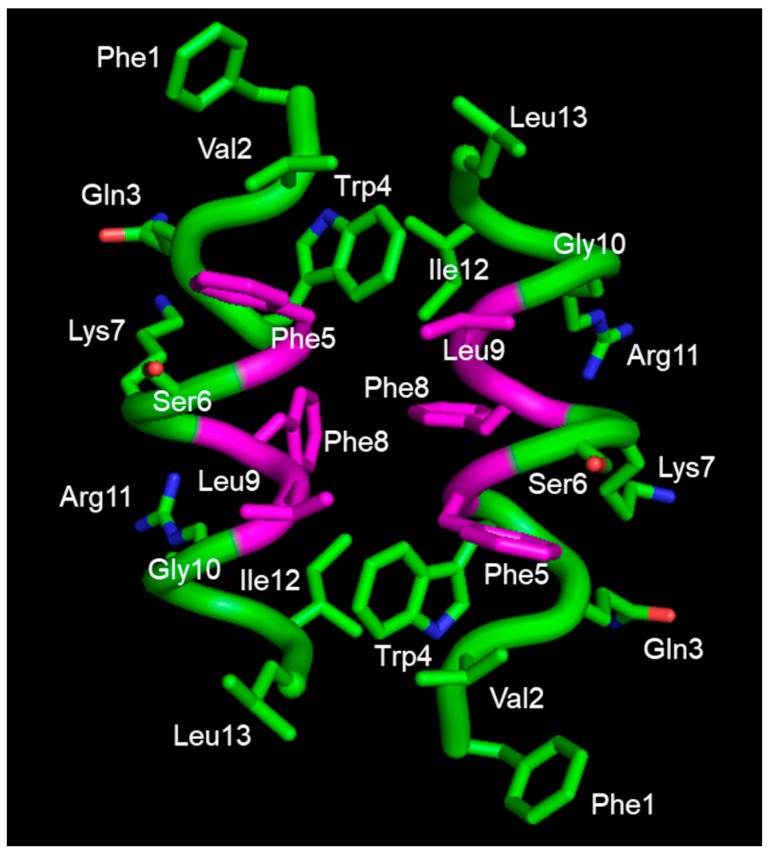
Structure of temporin L in LPS. Residues involved in dimer stabilization are indicated in magenta [67].

Using CD and NMR techniques, the interactions of temporin L with micelles of SDS and DPC, employed as mimic of bacterial and mammalian membranes, respectively, were investigated [58]. The peptide has a random coil structure in water and is an α-helix in SDS and dodecylphosphorylcholine (DPC) micelles. NMR in SDS reveals the existence of an α-helical structure encompassing residues 3 to 11, while the N and C-terminal ends appear less defined. In DPC, all residues are in an α-helix conformation. The topological orientation of the peptide in the micelles was detected using paramagnetic probes and NMR: in SDS, it is located at the liquid–water interface, while in DPC, it is perpendicular to the micelle surface with its N-terminus buried in the hydrophobic core of the micelle and the C-terminus in the water layer. These results are consistent with the experimentally observed tendency of temporin L to interact with mammalian membranes, such as those of erythrocytes. Fluorescence experiments confirmed the ability of temporin L to interact with *E. coli* LPS with a calculated dissociation constant of 4.4 µM, and suggest the formation of an antiparallel dimer [67]. NMR studies of temporin L in LPS micelles indicate that the peptide assumes an amphipathic α-helical structure, showing the hydrophobic and aromatic residues Trp4, Phe 5, Leu9, Phe8 and Ile12 on one face, and the hydrophilic residues Gln3, Ser6, Lys7 and Arg11 on the other face. Interactions between the residues of the non-polar face stabilize the dimer (Figure 2). CD studies carried out for the peptide in the presence of *E. coli* cells reveal that this peptide, as some analogues of temporin B, folds into a helix upon interaction with cells. CD spectra recorded on a mixture of temporin L with *S. epidermidis* cells suggest the folding of the peptide in a helical conformation, although signals are not very intense [89].

The ability of the peptide to permeate the cytoplasmic membrane of *E. coli* cells was investigated by monitoring the leakage of cytoplasmic β-galactosidase: the peptide increases the permeability of the inner membrane at concentrations in which it shows significant bactericidal activity [90]. Using a triple-staining assay to detect contemporarily total, viable and membrane-perturbed bacteria, Mangoni and co-workers found that a sublethal concentration of temporin L alters the integrity of the bacterial membrane to a significant extent without triggering cell death, and induces modifications to the cell shape, as shown through SEM experiments.

Using a combination of molecular modelling and electrophysiology experiments such as patch–clamp, the interaction of temporin L with model membranes was investigated [91]. The structure of the peptide in model membranes is helical. The peptide conformational flexibility was investigated through molecular dynamic simulations in 1-palmitoyl-2-oleoyl-sn-glycero-3-phosphoethanolamine (POPE)/POPG and POPG; the N-terminus of the peptide appears to be endowed with high flexibility and is involved in the penetration of the membrane. The association of the peptide through aromatic hydrophobic residues, namely Phe1, Phe5 and Phe 8, occurs. Patch–clamp experiments in 1,2-diphytanoyl-*sn*-glycero-3-phospho-(1′-*rac*-glycerol)(DPhPG) membranes, used as a mimic of Gram-negative bacteria cytoplasmic membrane, suggest that temporin L has channel-like activity, forming pores with a size twice the diameter of a chloride ion.

A deeper investigation of the mechanism of action of temporin L on Gram-negative bacteria was carried out using a combination of functional proteomic, fluorescence, docking, TEM, DLS and small-angle neutron scattering (SANS) to visualize the effect of the peptide on *E. coli* cells [92]. Initial studies allowed the identification of proteins belonging to the divisome as interactors of temporin L. The analysis of the morphology of bacteria incubated with the peptide revealed the formation of elongated structures, typically formed when cell division is impaired. Molecular docking, fluorescence experiments and biochemical assays supported the hypothesis that the protein FtsZ, responsible for the first step of bacterial cell division, is the target of temporin L. SANS analyses, aimed at investigating the effect of the peptide on *E. coli* cells focusing on structures formed in the range 2 to 300 nm, indicate that there is no difference in the lamellar structure of *E. coli* upon treatment with the peptide, which means no breaks in the membrane, and suggest a change in the spatial arrangement of the proteins targeted by the peptide. Overall, the mechanism of action appears to be the following: the peptide crosses the outer membrane and binds to FtsZ, triggering the inhibition of cell division and cell death [85,93,94].

#### 2.5.3. Temporin L Analogues

Many temporin L analogues were synthesized with the aim of reducing its cytotoxicity and, of course, improve its antimicrobial activity. Selected analogues will be described. Several studies started from the analysis of the structure of the peptide and investigated the relationship between the structure and the biological activity of the peptide. In 2008, in a study carried out in parallel on temporin A and temporin L, Carotenuto and co-workers hypothesized that the ability of temporin L to interact with mammalian membranes, therefore being cytotoxic, was related to its regular helical structure. With the aim of inducing a turn in the structure of the peptide, Gln3 was replaced by a proline; the resulting peptide Pro3TL showed increased activity against Gram-positive bacteria and yeast cells, and also reduced haemolytic activity with respect to the parent peptide [58]. The analysis of the structure of this peptide in SDS and DPC revealed a stable helical conformation between residues 6 and 13 and a flexible N-terminus, supporting the initial working hypothesis. The substitution of L-Leu9 by the D enantiomer yields a peptide devoid of haemolytic activity, showing improved activity against *C. albicans* and reduced activity against bacteria as compared to Pro3TL [95]. The structure of this peptide in SDS and DPC is a kinked helix. The structure is stabilized by an unusual *i* to *i* + 5 hydrogen bond between Phe5 and Gly10. This peptide shows anti-inflammatory activity in zymosan-induced peritonitis in in vivo models of mice [96]. In addition, the peptide is active against enveloped viruses [85]. The introduction of ornithine in position 11 of Pro3TL in the place of Leu11, or the replacement of Pro3 by 4-amino-proline resulted in a peptide with further reduced haemolytic activity and antimicrobial activity similar to that of Pro3TL [97,98].

The analogue of temporin L with norleucine (Nle) in position 1 and two more substitutions (NLe1, DLeu9, DLys10) differs from all the others in terms of structure; it is not a helical peptide. CD data suggest the formation of beta sheet structures in the presence of 1,2-dioleoyl-sn-glycero-3-phospho-(1′-rac-glycerol) (DOPG)/cardiolipin (CL) and DOPG/1,2-Dioleoyl-sn-glycero-3-phosphoethanolamine (DOPE) LUV. The increase in the hydrophobicity of the peptide due to the presence of NLe was related to an increased anti-inflammatory activity of the peptide in murine macrophage cell lines [99].

Srivastava and co-workers identified a phenylalanine zipper-like motif in temporin L. To determine the role of this structural motif, analogues in which phenylalanines in positions “a” and “d” of the zipper motif were replaced by alanine or leucine residues were obtained. It was found that the phenylalanine zipper motif affects the ability of the peptide to interact with LPS [100]. The introduction of leucines in place of phenylalanines in the analogue F5,8L TempL results in the introduction of a leucine zipper motif, which leads to increased LPS binding activity, increased ability of these peptides to dissociate LPS aggregates and, finally, enhanced endotoxic activity of the peptide. This peptide exhibits an alpha helical structure in the presence of LPS, with a percentage of helicity higher than temporin L. The haemolytic activity of the double-substituted F5,8L TempL is higher than temporin L, while the antibacterial activity is not improved. Peptides F5L TempL and F8L Temp L show moderately reduced haemolytic activity as compared to temporin L. In contrast, the replacement of phenylalanine by alanine impairs the ability of the peptide to bind to and fold in the presence of LPS, and reduces the production of LPS-induced pro-inflammatory cytokines. Interestingly, the peptide F5,8L TempL shows drastically reduced haemolytic and antibacterial activities.

Another interesting analogue of temporin L was designed by Kumari et al., starting from the analogue Q3K, which shows potent antiendotoxic activity and cytotoxicity comparable to the natural temporin L, changing the phenylalanine residues located at the hydrophobic face of the peptide (F8) and in the “d” position of the phenylalanine zipper by the cationic lysine residue and swapping the tryptophan residue W4 with serine S6. The analogue, named SW, Q3K, F8K, shows improved antimicrobial activity, comparable antiendotoxic activity and reduced haemolytic activity as compared to temporin L [101]. The low cytotoxicity and antimicrobial activity were also demonstrated in mice challenged with lethal doses of *P. aeruginosa*. This peptide is amphipathic and has clusters of hydrophobic and hydrophilic amino acids, resulting in a disordered secondary structure in bacterial membrane mimetic lipid vesicles. Overall data reported by Kumari and co-workers suggest new directions for the design of noncytotoxic antimicrobial peptides, highlighting the importance of a high hydrophobic moment together with a low secondary structure content.

## 3. Temporins from *Hylarana*

Peptides produced by four frogs belonging to the *Hylarana* family have been isolated. Limited data are available (Table 7); for example, in the case of temporin PTa from *Hylarana picturata*, we know that it is a 14-amino-acid peptide and that it is active against both Gram-positive bacteria, such as *S. aureus*, and Gram-negative bacteria, such as *E. coli* [102]. More information is available on peptides produced by *Hylarana guentheri*, named temporin GHa, GHb, GHc and GHd.

Temporins GHa to GHd were identified by analysing the sequence of cDNA isolated from *Hylarana guentheri*, a frog living in South China [103]. These are 13-amino-acid peptides, lacking positively charged residues such as arginine or lysine. According to secondary structure predictions, all peptides but temporin GHb show a certain degree of helicity. They have a broad spectrum of antimicrobial activity, being able to kill both Gram-positive and Gram-negative bacteria, including the methicillin-resistant *S. aureus*, and fungi. The activity of these peptides is independent of salt concentrations and remains almost unaltered when they are incubated with human serum and *S. aureus* V8 protease. The analysis of the morphology of different strains of *E. coli* and *S. aureus* treated with peptides suggests that peptides likely act by perturbing the cell membrane.

### Temporin GHa Analogues

Analogues of temporin GHa were obtained by replacing histidines in position 4 or 11 or both with lysines [102]. The peptide temporin GHaK with both histidines replaced shows increased activity against the tested strains of Gram-positive and Gram-negative bacteria. In addition, these peptides prevent the adhesion and formation of biofilms by *S. aureus.* Bacterial membrane permeability was increased upon incubation with the peptide. In a recent work, arginines were introduced in sequences to replace histidines in position 4 and 11 to give GHaR, as well as amino acids in positions 6, 7 and 8 to give temporins GHaR6R, GHaR7R, GHaR8R and GHaR9R [102]. The secondary structure of these peptides is predicted to be alpha helical. An analogue of temporin GHaR named GHaR9W, in which Leu9 was replaced by tryptophan, was also obtained. In general, the introduction of positive charges causes an increase in the antimicrobial activity of the peptides; all derivatives are more active than temporin GHa. Haemolytic activity appears to be high for GHaR and reduced in GHaR6R. SEM analyses revealed that these peptides severely perturb the membrane of *S. mutans*.

Temporin GHaR analogues show potent activity against MRSA, permeabilizing its cytoplasmic membrane, and are able to inhibit MRSA biofilm formation and eradicate mature biofilms. Interestingly, these peptides trigger the downregulation of the expression of the genes icaADBC involved in biofilm formation [105].

## 4. Temporins from *Lithobates*

Several temporins from the *Lithobates* genera have been investigated so far; data on the biological activities of these compounds are reported (Table 8), but to the best of our knowledge, their mechanism of action is not known. For example, temporins 1Ca to 1Ce isolated from skin extracts of *Rana clamitans* are 13-amino-acid peptides [106]. The analysis of their antimicrobial activity reveals that these peptides are active against *S. aureus*, and not against *E. coli* and *C. albicans*.

The temporin 1ARa isolated from skin secretions of *Lithobates areolata* is active against *S. aureus*. At high concentrations, it is active against *E. coli* and is inactive against *C. albicans* [107]. It shows haemolytic activity (HC_50_) at 210 µM concentration on human erythrocytes. No structure or mechanism has been reported so far.

Temporins CPa and CPb are secreted by the gopher frog *L. capito* [109]. These are 13-amino-acid peptides. The temporin CPa has an atypical primary structure (IPPFIKKVLTTVF-NH_2_) and activity, being more active against Gram-negative than Gram-positive bacteria. Haemolytic activity measured against human erythrocytes as HL50 is >120 µM. Studies on the anticancer activity of these peptides carried out using mouse breast cancer cells (4T1) indicate that LC50 were >80 µM.

## 5. Temporins from *Odorrana*

Skin secretion of *Odorrana hainanensis* contains peptides belonging to the temporin family, namely temporin HN1 (AILTTLANWARKFL-NH_2_) and HN2 (NILNTIINLAKKIL-NH_2_) [110]. Both contain 14 amino acids and two positively charged amino acids. These peptides are mainly active against Gram-positive bacteria and fungi such as *C. albicans*, with temporin HN2 being the most active. Unlike other temporins, these are poor in leucines and contain alanines (Table 9). The haemolytic activity of these peptides is weak. According to predictions, these temporins should have an α-helical structure. The Chinese bamboo leaf odorous frog *Odorrana versabilis* secretes many antimicrobial peptides, including temporin 1VE [111]. Other than the sequence, there is no information on its activity or structure.

## 6. Temporins from *Pelophylax*

Studies on temporin secreted by frogs of the *Pelophylax* family are mainly focused on peptides secreted by the *Pelophylax saharica*.

### 6.1. Temporin SH Family

Peptides belonging to the family of temporin SH were identified in the skin of the North African frog *Pelophylax saharica* [112]. Currently, six members have been identified and characterized (see Table 1). They present a length ranging from 8 to 17 residues with different net charges. These peptides are active against Gram-positive and Gram-negative bacteria, yeasts and fungi with MIC values below 30 µM (see Table 10). They also show activity against *Leishmania*, *L. pneumophila* and *Helicobacter pylori*. In some cases, anticancer activity was detected. Peptides Temporin SHa and SHf are the most interesting members of this family.

#### 6.1.1. Temporin SHa Biological Activity

Temporin SHa presents a net charge of 2+, a broad-spectrum of activity and high potency against Gram-negative and Gram-positive bacteria (Table 10). It is particularly active against *E. coli* and *P. aeruginosa* (MIC = 31 µM). The haemolytic activity, measured as LC_50_, is 25 µM. Temporin SHa shows high activity against clinical strains of *H. pylori* and no toxicity on human gastric cells in vitro and on human gastric explants. Its activity is not reduced in the presence of mucins or human gastric mucus. Instead, the presence of pepsin prevents temporin SHa activity [117]. The peptide is active against several species of *Legionella*, with MIC values ranging from 3 to 6 μM. Experiments on *L. pneumophila* cells showed that the SHa peptide induces cell permeabilization, which would mainly account for the bactericidal activity of this peptide. Temporin SHa is able to reduce the amount of *L. pneumophila* inside eukaryotic cells, inducing a host response, as it is able to penetrate into the cytoplasm but is not able to enter vacuoles; for these reasons, it protects the bacteria from the action of the peptide [118].

Furthermore, it showed antiparasitic activity (IC_50_ = 20 µM) against the promastigote and amastigote stages of *Leishmania infantum* and *Leishmania Mexicana* (4 and 42 μM, respectively) [119]. The difference in activity was attributed to the different composition of the membrane of the two parasite forms. Specifically, the peptide temporin SHa interacts with the proteophosphoglycans present on the surface of the cell in the promastigote form but absent in the cell surface of the amastigote stage [119]. Temporin SHa shows anti-viral activity against herpes simplex virus type 1, directly acting on the virus (virucidal activity) instead of eliciting an immunomodulatory response [120]. Temporin SHa shows anticancer activity against specific cancer cell lines such as MCF-7 (human breast cancer; IC_50_ = 14.5 μM), HeLa (IC_50_ = 18.4 μM) and NCI-H460 (NSLC; IC_50_ = 34.5 μM).

#### 6.1.2. Temporin SHa Structure and Mechanism of Action

**Figure 3:**
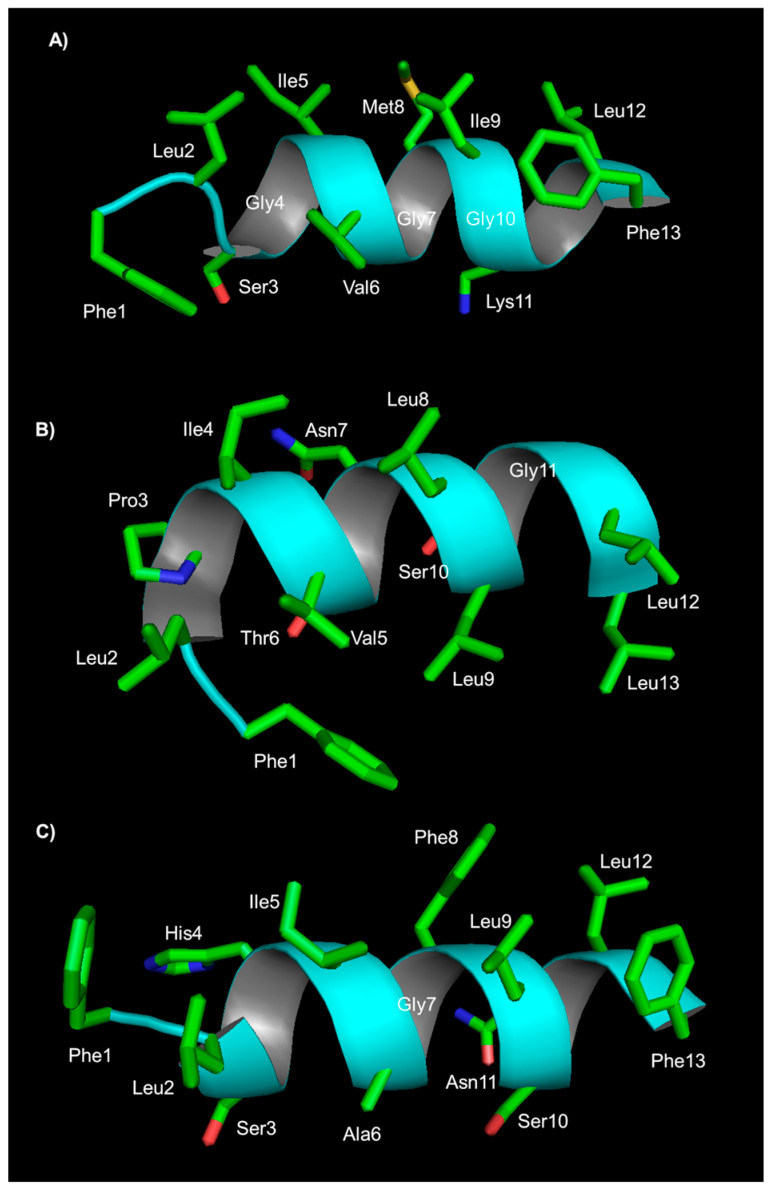
NMR structure in SDS of peptide (**A**) temporin SHa, (**B**) temporin SHb and (**C**) temporin SHc [113].

The NMR structure of temporin SHa was determined in 80 mM SDS. In water, a small amount of secondary structure is present. In the presence of SDS, the peptide adopts an amphipathic helical secondary structure involving residues 3 to 12 (Figure 3). Analysis with paramagnetic probes to detect the position of the peptide within the micelle suggests that temporin SHa adopts a nearly parallel orientation with respect to the micelle surface. Binding studies with multilamellar vesicles suggest a binding mode based on in-plane insertion into the lipid bilayer [113]. The decrease in hydrophobicity, replacing large aliphatic residues (leucine and valine) with alanine in the [A^2,6,9^]SHa analogue peptide, reduces the ability of the peptide to fold in an α-helical structure in the presence of the membrane model system, and prevents antimicrobial activity, suggesting a primary role of the interaction between he hydrophobic side chains and lipid acyl chains.

#### 6.1.3. Temporin SHf Biological Activity

Another interesting and peculiar member of the temporin SH family is the peptide temporin SHf. It is a short 8-mer peptide rich in the aromatic residue phenylalanine [116]. It shows broad antimicrobial activity against Gram-positive and Gram-negative bacteria and yeasts. Exceptions are *E. coli* D21, *E. coli* ATCC 35218, *P. aeruginosa* and the fungus *Aspergillus flavus*. No activity was detected against the promastigote and axenic amastigote forms of *Leishmania infantum*. Temporin SHf is not cytotoxic against human erythrocytes (LC_50_ = 200 µM) and has no haemolytic activity at a 50 μM concentration.

#### 6.1.4. Temporin SHa Structure and Mechanism of Action

Structural analysis of temporin SHa in DPC micelles reveals that the peptide adopts an α-helix conformation involving residues 3 to 8, with the peptide interacting with the surface of the micelle and inserting Phe 3, Leu 4 and Phe 8 residues inside the micelle [116]. The mechanism of action consists of membrane permeabilization with release of the intracellular content. Calorimetric studies carried out in the presence of 1,2-dimyristoyl-sn-glycero-3-phosphocholine (DMPC), a model for mammalian cell membranes, or dimyristoyl phosphatidylglycerol (DMPG), a model for negatively charged bacterial membrane, suggest that the peptide temporin SHf interacts strongly and exclusively with DMPG, indicating a selectivity towards the bacterial cell membrane.

#### 6.1.5. Temporin SH Analogues

Several analogues of temporin SHa were obtained by introducing D-alanine in the place of amino acids in position 4, 7 and 10 to give [G4a]-SHa, [G7a]-SHa and [G10a]-SHa peptides. These analogues were tested, and their mechanism of action evaluated against methicillin-resistant *S. aureus* [121]. [G10a]-SHa showed an MIC comparable with the natural peptide (3.58 vs. 3.62 μM), but performed better in physiological conditions such as 30% foetal bovine serum, 30% human serum and 10% human blood (in these conditions, the natural peptide was unstable). In addition, [G10a]-SHa is very active against clinical strains of *H. pylori* and maintains its activity after treatment with pepsin [117]. However, it has a more pronounced cytotoxicity and haemolytic profile with respect to the parent and other analogue peptides. Overall, the [G10a]-SHa peptide seems to be more suitable for skin applications. Atomic force microscopy (AFM) experiments showed that the mechanism of action of these peptides consists of membrane rupturing, triggering leakage of cytoplasmic content. [G10a]-SHa is cytotoxic to NIH-3T3 murine fibroblast cell line and more active than temporin SHa against the indicated cancer cell lines. The conjugation of [G10a]-SHa with a breast cancer-targeting peptide furnishes a hybrid peptide with no cytotoxicity towards non-cancer cells but still active against MCF-7 cell lines [122].

The peptide [K3]SHa contains a lysine in place of a serine in position 3; it is non-haemolytic and it is more active than the parent peptide against several Gram-positive and Gram-negative bacterial strains, including multidrug-resistant strains of the clinically relevant pathogenic species *S. aureus*. This peptide kills bacteria and induces a potent membrane permeabilization/depolarization, likely through a detergent-like mechanism. The improved antimicrobial activity with respect to the parent peptide could be, in part, ascribed to a stronger interaction with the anionic membrane. Temporin [K3]SHa retains the ability to fold in an α-helical structure in the presence of membrane mimetic due to enhanced electrostatic interaction [123]. Interestingly, temporin [K3]SHa shows antifungal activity, with an MIC in the range 5.5–45 μM. In particular, it acts rapidly against *C. albicans*, inducing permeabilization of the membrane [124]. It also shows leishmanicidal activity, being more potent than temporin SHa. A growth inhibitory effect was observed with promastigotes of the *Leishmania infantum*, *Leishmania major, Leishmania amazonensis* and *Leishmania braziliensis* species, with amastigote forms of *L. infantum* and other trypanosomatids, such as *Trypanosoma brucei gambiense* and *Trypanosomacruzi* [123]. The anti-*Leishmania* activity was also analysed; it is mainly due to a membranolytic mechanism at peptide concentrations above IC_50_, whereas below IC_50_, it can induce apoptosis-like cell death. Finally, temporin [K3]SHa shows anti-viral activity [120].

Several temporin SHf analogues were designed in order to improve the antimicrobial activity without increasing the haemolytic activity [125]. Structure analysis studies revealed that the analogue [p-tBuF2, R5]SHf, presenting the unnatural amino acid p-*ter*butyl-phenylalanine in position 2 and arginine in position 5, retains the helical conformation in the presence of SDS and shows improved antimicrobial activity with respect to the natural peptide, being also active against Gram-negative bacteria such as *E. coli* ATCC 25922 at physiological salt concentration (150 mM NaCl) and in 30% serum.

## 7. Biomedical/Biotechnological Applications of Temporins

Applications of temporins as drugs are still limited, likely due to their quick in vivo degradation. The formulation of temporins into liposomal vectors or otherwise has been reported [126]. Some examples will be reported here. The encapsulation of temporin B into chitosan nanoparticles was described; encapsulation of the peptide reduces its cytotoxicity against mammalian cells. Sustained antibacterial activity was demonstrated against various strains of *S. epidermidis*; the gradual release of the peptide results in a reduction in viable bacterial count and inhibition of the regrowth of residual cells [126]. The use of temporins for topical applications to combat *Candida* infections was recently hypothesized [77]. Studies aimed at formulating the peptide temporin L have been reported. In 2020, Brancaccio and co-workers described the use of cyclodextrins as tools to improve the pharmacokinetic profile and reduce the toxicity of temporin L. Cyclodextrins complexed to temporin L were demonstrated to effectively hamper the attachment of biofilms produced by clinical isolates of *P. aeruginosa* and MRSA and eradicate bacteria. The enzyme-triggered release of a caged temporin L was proposed and tested in HEK293T cells [94].

The analogue of temporin L named [Pro^3^]Temporin L was encapsulated in poly(2-hydroxyethyl methacrylate) microgel particles to improve delivery and control the peptide release. The analysis of antibacterial activity against *S. aureus* showed that encapsulation was effective; the peptide maintains its biological activity and a sustained release of the peptide was observed [127].

More recently, L-K6, an analogue of temporin 1CEa, was embedded in a liposome presenting on its surface folic acid as a cancer-targeting agent and evaluated in vitro and in vivo on human breast cancer MCF-7 cells [128]. The peptide was selectively targeted to cancer cells, and tumour growth was reduced by about three times in ten days of observation. Even though L-K6 was able to selectively target MCF-7 cancer cells, the use of a decorated liposome was needed to reduce enzymatic degradation [31].

Peptides can be immobilized on solid surfaces to produce antimicrobial materials, which is useful for the production of implantable materials [129]. Temporins SHa, [K^3^]SHa and D-[K^3^]SHa were covalently linked on a gold surface; immobilized peptides retain antimicrobial activity against *Listeria ivanovii*. Immobilization was performed by exploiting amide bond formation, reacting either the amin of the amino terminus or lysine side chains, or the carboxy C-terminus of a modified peptide. The surface decorated with temporin D-[K^3^]SHa showed effective antiadhesive properties reducing the bacterial adhesion to the modified gold surface by up to 50%. Immobilization through the C-terminal end was the most efficient [130,131]. A strategy to immobilize temporin SHa on a titanium surface was reported [132]. Chemoselectively immobilized temporin SHa on titanium surfaces showed the ability to inhibit the growth of *E. coli* and *S. epidermidis*. Better results are achieved when the peptide is grafted through a central lysine residue instead of via the N- and C-termini. In a different report, the temporin B analogue TB_KKG6A was immobilized onto microcrystalline cellulose; the functionalization of the cellulose confers antimicrobial activity against *E. coli* cells to the polymer [133].

Temporin A associated through electrostatic interactions with the enzyme organophosphorus hydrolase was found to overcome bacterial resistance acting on the quorum sensing mechanism mediated by N-acyl homoserine lactones. This nanocomplex showed improved antibacterial activity with respect the peptide alone when tested against *Bacillus subtilis* and *Pseudomonas* sp. [134]. Successively, this nanocomplex was entrapped in poly(vinyl alcohol) cryogel (PVA-CG)/bacterial cellulose material, forming a composite antibacterial material or bacterial cellulose [135,136].

*Rana chensinensis* skin peptides with antimicrobial properties including peptides belonging to the temporin 1C family were used to prepare different biomaterials for wound healing application. Composite nanofibres, loaded with peptides, were prepared through coaxial electrospinning of polyvinylpyrrolidone and sodium alginate, and through uniaxial electrospinning of polyvinylpyrrolidone and polycaprolactone also loaded with silver nanoparticles [137,138]. In vitro and in vivo experiments showed that the nanofibres release the bioactive compounds, are biocompatible and promote wound healing. Hybrid microspheres were also prepared using a polyvinyl alcohol gelatine hydrogel matrix and sodium alginate (SA) frog egg-like microspheres. This system can deliver *Rana chensinensis* skin peptides and induces wound repair in vivo [139].

Temporins have also been exploited to produce biosensors. Temporin PTa was implemented in an electrochemical biosensor for the detection and differentiation of bacterial strains [140]. The sensor is based on a gold electrode. The transducing layer contains a monolayer of mercaptobenzoic acid and gold-capped magnetic nanoparticles (Fe_3_O_4_Au), decorated with 3-(aminopropyl)triethoxysilane and finally derivatized with the peptide. Analysis is performed through electrochemical spectroscopy impedance and cyclic voltammetry. The sensor detects Gram-negative bacteria such as *K. pneumoniae* and *A. baumannii* with a limit of 10 CFU/mL and discriminates other organisms such as *B. subtilis* and *C. albicans*, exploiting structural differences in the bacterial membranes.

Temporin L was used as a membrane-interacting domain to develop a PET probe for in vivo imaging of granzyme B activity. Enzymatic cleavage allows radiolabelled temporin L to assume a helical conformation and insert into adjacent cellular membranes [141].

## 8. Conclusions

Antimicrobial peptides belonging to the temporin family have been isolated from the skin of frogs from all over the world or have been identified from cDNA libraries of skin secretions. Irrespective of the geographical location of the frog or the frog genus, temporin sequences are rich in leucines and isoleucines, while lacking cysteine. Most of the temporins contain a single positively charged amino acid (typically one lysine). These features determine their 3D structure and their antimicrobial activity. While most temporins effectively kill Gram-positive bacteria, some, such as temporin L from *Rana temporaria* or temporin CPa from *L. capito*, show strong activity against Gram-negative bacteria. Most temporins have no preferential structure in buffer and assume a helical structure in membrane-mimicking environments (e.g., in POPG vesicles or DOPG/PC mixtures) or in the presence of components of the bacterial outer membrane (such as LPS). The formation of beta-sheet structures has been hypothesized for temporin Rb in DOPG/DOPE/CL LUV based on CD analysis [101]. The interactions of peptides with the bacterial outer membrane have been demonstrated in many cases using fluorescence microscopy techniques, while intracellular targets have been identified in only a few cases. Due to their short sequences, these peptides are ideal candidates for structure–activity studies and, therefore, an ideal starting point to produce new and more active compounds. Changes in the peptide primary structure have been related to increases in their antimicrobial or haemolytic activity; in particular, many studies demonstrate that an increase in the positive charges of peptides translates into an increase in activity against Gram-negative bacteria.

In consideration of the growing interest of the scientific community in these molecules, based on the exponential increase in published papers on temporins and their analogues, in the near future, we expect to see a deeper exploitation of temporins in technological and medical applications.

## Figures and Tables

**Table 1:** Temporin sequences divided by frog genera as reported in the DBAASP database and recent reviews [15,17].

Genus	Specie	Peptide Name	Sequence ^a^
*Amolops*			
	*A. chunganensis*	Temporin CG1	FLPFVGNLLKGLL
		Temporin CG2	FFPIVGKLLSGLF
		Temporin CG3	FLPIVGKLLSGLF
		Temporin CG4	FLPILGNLLNGLL
		Temporin CG5	FLPFVGNLLNGLL
	*A. daiyunensis*	Temporin DY1	FFPMLADLVSKIF
	*A. jingdongensis*	Temporin AJ1	FLPIVTGLLSSLL
		Temporin AJ2	FFPIGGKLLFGLL
		Temporin AJ3	FFPIVGKLLSGLL
		Temporin AJ4	FFPIVGKLLFGLL
		Temporin AJ5	FPPIVGKLLFGLL
		Temporin AJ6	FLPIVGKLLSGLL
		Temporin AJ7	FLPIVGKLPSGLL
		Temporin AJ8	FFPIVGKRLYGLL
		Temporin AJ9	FLPIVGKLLSGLTGLL
		Temporin AJ10	FLPIVGKLLSGLSGLL
		Temporin AJ11	FFPIGGKLLSGLTGLL
	*A. lifanensis*	Temporin LF1	FLPFVGKLLSGLL
	*A. loloensis*	Temporin ALa	FLPIVGKLLSGLSGLL
		Temporin ALb	FLPIVGKLLSGLL
		Temporin ALc	LLPIVGKGLSGKL
		Temporin ALd	FLPIAGKLLSGLSGLL
		Temporin ALe	FFPIVGKLLFGLSGLL
		Temporin ALf	FFPIVGKLLSGLSGLL
		Temporin ALg	FFPIVGKLLFGLFGLL
		Temporin ALh	FLPIVGKLLSGLSGLS
		Temporin ALi	FFPIVGKLLSGLL
		Temporin ALj	FFPIVGKLLFGLL
		Temporin ALk	FFPIVGKLLS
*Euphlyctis*			
	*E. cyanophlyctis*	Temporin ECa	FLPGLLAGLL
*Hylarana*			
	*H. guentheri*	Temporin GH	FLPLLFGAISHLL
		Temporin GHa	FLQHIIGALGHLF
		Temporin GHb	FIHHIIGALGHLF
		Temporin GHc	FLQHIIGALTHIF
		Temporin GHd	FLQHIIGALSHFF
		Temporin GUa	FLQHIIGALSHIF
		Temporin GUb	FLPLLFGAISHIL
		Temporin GUc	FFPLIFGALSSILPKIL
	*H. latouchii*	Temporin LT1	FLPGLIAGIAKML
		Temporin LT2	FLPIALKALGSIFPKIL
		Temporin LTa	FFPLVLGALGSILPKIF
		Temporin LTb	FIITGLVRGLTKLF
		Temporin LTc	SLSRFLSFLKIVYPPAF
		Temporin LTe	FLAGLIGGLAKML
		Temporin HLa	FFPLIFGALSSILPKIL
		Temporin HLb	FLQHIIGALSHIF
	*H. picturata*	Temporin PTA	FFGSVLKLIPKIL
	*H. taipehensis*	Temporin TP1	FLPVLGKVIKLVGGLL
		Temporin TP2	FLPLLVGAISSILPKIF
		Temporin TP3	FLPLLFGALSTLLPKIF
	*H. spinulosa*	Temporin SN1	FFPFLLGALGSLLPKIF
		Temporin SN2	FITGLIGGLMKAL
		Temporin SN3	FISGLIGGLMKAL
		Temporin SN4	FITGLISGLMKAL
		Temporin SN5	FFPLVLGALGSILPKIF
	*H. maosonensis*	Temporin MS1	FLTGLIGGLMKALGK
		Temporin MS4	FLSGLIGGLAKMLGK
*Limnonectes*			
	*L. kuhlii*	Temporin LK1	FFPLLFGALSSMMPKLF
*Lithobates*			
	*L. areolatus*	Temporin 1AR	FLPIVGRLISGLL
	*L. capito*	Temporin CPa	IPPFIKKVLTTVF
		Temporin CPb	FLPIVGRLISGIL
	*L. catesbeianus*	Temporin La	LLRHVVKILEKYL
	*L. clamitans*	Temporin 1Ca	FLPFLAKILTGVL
		Temporin 1Cb	FLPLFASLIGKLL
		Temporin 1Cc	FLPFLASLLTKVL
		Temporin 1Cd	FLPFLASLLSKVL
		Temporin 1Ce	FLPFLATLLSKVL
	*L. grylio*	Temporin 1Ga	SILPTIVSFLSKVF
		Temporin 1Gb	SILPTIVSFLSKFL
		Temporin 1Gc	SILPTIVSFLTKFL
		Temporin 1Gd	FILPLIASFLSKFL
	*L. heckscheri*	Temporin 1HKa	SIFPAIVSFLSKFL
	*L. okaloosae*	Temporin 1OLa	FLPFLKSILGKIL
		Temporin 1OLb	FLPFFASLLGKLL
	*L. onca*	Temporin ONa	FLPTFGKILSGLF
	*L. palustris*	Temporin 1PLa	FLPLVGKILSGLI
	*L. pipiens*	Temporin 1P	FLPIVGKLLSGLL
	*L. septentrionalis*	Temporin 1SPa	FLPLVGKILSGLI
		Temporin 1SPb	FLSAITSLLGKLL
		Temporin 1SPc	FLSAITSILGKLF
	*L. sphenacephalus*	Temporin 1S	LLFGKIISRLLGN
	*L. virgatipes*	Temporin 1Va	FLSSIGKILGNLL
		Temporin 1Vb	FLSIIAKVLGSLF
		Temporin 1Vc	FLPLVTMLLGKLF
*Odorrana*			
	*O. hainanensis*	Temporin HN1	AILTTLANWARKFL
		Temporia HN2	NILNTIINLAKKIL
	*O. versabilis*	Temporin 1VE	FLPLVGKILSGLI
*Pelophylax*			
	*P. esculentus*	Temporin PE	FLPIVAKLLSGLL
		Temporin 1Re	FLPGLLAGLL
		Temporin 1Ec	FLPVIAGLLSKLF
		Temporin 1Ee	FLPVIAGVLSKLF
	*P. fukienensis*	Temporin PF	FLPLIAGLFGKIF
	*P. hubeiensis*	Temporin HB1	FLPLLAGLAAKWF
		Temporin HB2	FLPFLAGLFGKIF
	*P. nigromaculatus*	Temporin 1KM	FIPLVSGLFSRLL
		Temporin 1GY	VIPIVSGLLSSLL
	*P. ridibundus*	Temporin Ra	FLKPLFNAALKLLP
		Temporin Rb	FLPVLAGVLSRA
	*P. saharicus*	Temporin SHa	FLSGIVGMLGKLF
		Temporin SHb	FLPIVTNLLSGLL
		Temporin SHc	FLSHIAGFLSNLF
		Temporin SHd	FLPAALAGIGGILGKLF
		Temporin SHe	FLPALAGIAGLLGKIF
		Temporin SHf	FFFLSRIF
*Rana*			
	*R. aurora*	Temporin 1AUa	FLPIIGQLLSGLL
	*R. boylii*	Temporin 1BYa	FLPIIAKVLSGLL
	*R. cascadae*	Temporin 1CSa	FLPIVGKLLSGLL
		Temporin 1CSb	FLPIIGKLLSGLL
		Temporin 1CSc	FLPLVTGLLSGLL
		Temporin 1CSd	NFLGTLVNLAKKIL
	*R. chensinensis*	Temporin 1CEa	FVDLKKIANIINSIF
		Temporin 1CEb	ILPILSLIGGLL
		Temporin 1CEc	IIPLPLGYFAKKT
		Temporin 1CEd	ILPLIASLIGGLL
		Temporin 1CEe	ILPIIGKILSTIFGK
		Temporin 1CEf	ILPILGKILSTIL
		Temporin 1CEg	ILPIFSWIGHLF
		Temporin 1CEh	FVDLKKIANILNSIF
	*R. draytonii*	Temporin 1DRa	HFLGTLVNLAKKIL
		Temporin 1DRb	NFLGTLVNLAKKIL
		Temporin 1DRc	FLPIIASVLSSLL
	*R. dybowskii*	Temporin 1DYa	FIGPIISALASLFG
		Temporin CDYa	LPLVGNLLNDLL
		Temporin CDYb	ILPILSLIGGLL
		Temporin CDYc	VLPLVGNLLNDLL
		Temporin CDYd	FIGPLISALASLFKG
		Temporin CDYe	FIGPIISALASLFGG
	*R. italica*	Temporin ITa	FLGAIAQALTSLLGKL
	*R. japonica*	Temporin 1Ja	ILPLVGNLLNDLL
	*R. luteiventris*	Temporin 1La	VLPLISMALGKLL
		Temporin 1Lb	NFLGTLINLAKKIM
		Temporin 1Lc	FLPILINLIHKGLL
	*R. muscosa*	Temporin 1M	FLPIVGKLLSGLL
	*R. nigromaculata*	Temporin 1RNa	ILPIRSLIKKLL
		Temporin 1RNb	FLPLKKLRFGLL
	*R. nigrovittata*	Temporin RN1	FLPLVLGALSGILPKIL
		Temporin RN2	FFPLLFGALSSLLPKLF
		Temporin RN3	FFPLLFGALSSHLPKLF
	*R. ornativentris*	Temporin 1Oa	FLPLLASLFSRLL
		Temporin 1Ob	FLPLIGKILGTIL
		Temporin 1Oc	FLPLLASLFSRLF
		Temporin 1Od	FLPLLASLFSGLF
		Temporin 1Oe	ILPLLGNLLNGLL
		Temporin 1Of	SLLLKGLASIAKLF
		Temporin 1Og	FLSSLLSKVVSLFT
	*R. pirica*	Temporin 1PRa	ILPILGNLLNGLL
		Temporin 1PRb	ILPILGNLLNSLL
	*R. pretiosa*	Temporin PRa	FLPILGNLLSGLL
		Temporin PRb	FLPIITNLLGKLL
		Temporin PRc	NFLDTLINLAKKFI
		Temporin PRe	FLPLAMALGKLL
	*R. sakuraii*	Temporin 1Ska	FLPVILPVIGKLLNGIL
		Temporin 1SKb	FLPVILPVIGKLLSGIL
	*R. tagoi*	Temporin 1TGa	FLPILGKLLSGIL
		Temporin 1TGb	AVDLAKIANKVLSSLF
		Temporin 1TGc	FLPVILPVIGKLLSGIL
	*R. tagoi okiensis*	Temporin TOa	FLPILGKLLSGFL
		Temporin TOb	FLPILGKLLSGLL
	*R. temporaria*	Temporin A	FLPLIGRVLSGIL
		Temporin B	LLPIVGNLLKSLL
		Temporin C	ILPILGNLLNGLL
		Temporin D	LLPIVGNLLNSLL
		Temporin E	VLPIIGNLLNSLL
		Temporin F	FLPLIGKVLSGIL
		Temporin G	FFPVIGRILNGIL
		Temporin H	LSPNLLKSLL
		Temporin K	LPPNLLKSLL
		Temporin L	FVQWFSKFLGRIL
	*R. tsushimensis*	Temporin 1TSa	FLGALAKIISGIF
		Temporin 1TSb	FLPLLGNLLNGLL
		Temporin 1TSc	FLPLLGNLLRGLL
		Temporin 1TSd	FLPLLASLIGGML

^a^ C-terminal end is amidated.

**Table 3:** Antimicrobial activity against selected microorganisms of temporin A.

Microorganism	Antimicrobial Activity (µM)	Ref
*E. coli* D21	LC: 11.9	[37]
*S. aureus* Cowan 1	LC: 2.3	[37]
*C. albicans* ATCC 10261	LC: 3.4	[37]

LC: lethal concentration.

**Table 4:** Antimicrobial activity against selected microorganisms of peptide temporin B.

Microorganism	Antimicrobial Activity	Ref
*E. coli* ^a^	MIC: >100 μg/mL	[63]
*S. aureus* A170	MIC 100: 25 μg/mL	[63]
*C. albicans* ATCC 10261	LC: 4.0 μM	[37]

^a^ strain not indicated; MIC: minimum inhibitory concentration; LC: lethal concentration.

**Table 5:** Antimicrobial activity against selected microorganisms of peptide temporin G.

Microorganism	Antimicrobial Activity (µM)	Ref
*S. aureus* ^a^	BC: 24	[79]
*C. albicans* ATCC10231	MIC: 16	[80]

^a^ strain 1 as defined in [79]; BC: bactericidal concentration; MIC: minimum inhibitory concentration.

**Table 6:** Antimicrobial activity against selected microorganisms of peptide temporin L.

Microorganism	Antimicrobial Activity as MIC (µM)	Ref
*E. coli* D21	12	[58]
*S. aureus* ATCC 25923	3	[58]
*C. albicans* ATCC 10231	12	[58]

MIC: minimum inhibitory concentration.

**Table 7:** Antimicrobial activity against selected microorganisms of peptides from *Hylarana*.

Peptide	Antimicrobial Activity as MIC (µM)					
	*S. aureus* ATCC 25923	Ref	*E. coli* ATCC 25922	Ref	*C. albicans* ATCC 10231	Ref
Temporin GHa	6.8	[103]	26.6	[103]	26.6	[103]
Temporin GHb	6.8	[103]	13.2	[103]	26.4	[103]
Temporin GHc	12.9	[103]	104	[103]	104	[103]
Temporin GHd	12.7	[103]	12.7	[103]	25.5	[103]
Temporin PTa	3.12 ^a^	[104]	25 ^b^	[104]	n.d.	

MIC: minimum inhibitory concentration; the peptide was tested on ^a^: *S.aureus* USA300, ^b^: *E.coli* K12; n.d.: not determined.

**Table 8:** Antimicrobial activity against selected microorganisms of temporins from *Lithobates*.

Peptide	Antimicrobial Activity as MIC (µM)					
	*S. aureus*	Ref	*E. coli* ATCC 25922	Ref	*C. albicans*	Ref
Temporin 1Cb	140 ^a^	[106]	N.A.	[106]	N.A. ^c^	[106]
Temporin 1Cd	80 ^a^	[106]	N.A.	[106]	N.A. ^c^	[106]
Temporin 1Ce	100 ^a^	[106]	N.A.	[106]	N.A. ^c^	[106]
Temporin 1AR	15 ^a^	[107]	125	[107]	N.A. ^c,e^	[107]
Temporin CPa	≥25 ^b^	[108]	12.5	[108]	25 ^d^	[108]
Temporin CPb	12.5 ^b^	[108]	>50	[108]	50 ^d^	[108]

MIC: minimum inhibitory concentration; N.A.: not active at 150 μM; the peptide was tested ^a^: on *S. aureus* NCTC 8325, ^b^: on *S. aureus* USA300, ^c^: on *C. albicans* ATCC90028, ^d^: on *C. albicans* ATCC 10231, ^e^: at 200 μM.

**Table 9:** Antimicrobial activity against selected microorganisms of temporins from *Odorrana*.

Peptide	Antimicrobial Activity as MIC (µM)					
	*S. aureus* ATCC25923	Ref	*E. coli* ATCC25922	Ref	*C. albicans* ATCC2002	Ref
Temporin HN1	37.5	[110]	N.A.	[110]	75	[110]
Temporin HN2	4.8	[110]	N.A.	[110]	9.8	[110]

MIC: minimum inhibitory concentration; N.A.: not active at 150 μM.

**Table 10:** Antimicrobial activity against selected microorganisms of temporins SH.

Peptide	Antimicrobial Activity as MIC (µM)					
	*S. aureus* ATCC25923	Ref	*E. coli* ATCC25922	Ref	*C. albicans* ATCC90028	Ref
Temporin SHa	3	[113]	10	[113]	16	[113]
Temporin SHb	58	[113]	231	[113]	>116	[113]
Temporin SHc	10	[113]	>161	[113]	20	[113]
Temporin SHd	6.25	[114]	5	[114]	100	[114]
Temporin SHe	3.12	[115]	25	[115]	>100	[115]
Temporin SHf	12.5	[116]	25	[116]	50	[116]

MIC: minimum inhibitory concentration.
